# Cyanide poisoning after bitter almond ingestion: “A rare case report”

**DOI:** 10.1002/ccr3.8418

**Published:** 2024-01-08

**Authors:** Neda Arabizadeh, Masoud Mahmoudi, Laaya Mokhtar Gandomani, Nastaran Eizadi‐Mood

**Affiliations:** ^1^ School of Medicine Islamic Azad University Najafabad Branch Isfahan Iran; ^2^ Clinical Toxicology Department, School of Medicine, Isfahan Clinical Toxicology Research Center Isfahan University of Medical Science Isfahan Iran; ^3^ School of Medicine Isfahan University of Medical Science Isfahan Iran

**Keywords:** bitter almonds, cyanides, sodium nitrate, toxicology

## Abstract

We present a case of a 36‐year‐old woman with a history of three suicide attempts who had ingested approximately 40 bitter almonds in a suicidal act, leading to her admission to the emergency department of a regional hospital due to complaints of vomiting. Upon arrival, she exhibited confusion, and her vital signs were recorded as follows: pulse rate = 117 beats/min, blood pressure = 160/85 mmHg, oxygen saturation = 95%, respiratory rate = 16, temperature = 37°C. The patient venous blood gas analysis manifested severe metabolic acidosis (pH = 6.92, pO_2_ = 43 mmHg, HCO_3_ = 8.6 mmol/L, pCO_2_ = 42.7 mmHg, base excess = −25.9 mmol/L). Four hours later, she became unconscious and she was intubated. Gastric lavage and a single dose of 60 g of activated charcoal and sodium bicarbonate were administered. In the referral hospital, sodium nitrite was given due to the severity of the poisoning, and norepinephrine infusion was initiated to manage hypotension. Within a day, the patient regained consciousness, underwent extubation, and after 72 h was discharged and subsequently transferred to psychiatric care for further treatment. This case underscores the critical, life‐threatening implications of cyanide toxicity following the ingestion of bitter almonds, highlighting the efficacy of supportive measures such as gastric lavage, activated charcoal, and sodium bicarbonate. Furthermore, it emphasizes the successful application of sodium nitrite monotherapy in managing this condition.

## Graphical abstract

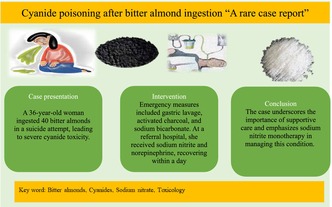

## 1 INTRODUCTION

Cyanide poisoning is a life‐threatening emergency that can occur by inhalation, ingestion, or skin absorption.^1^ Oral cyanide poisoning typically results from ingesting cyanogenic compounds found in certain plants such as peaches, apricots, and bitter almonds.^2^ These compounds can produce hydrogen cyanide, which can enter cells and inhibit cellular respiration.^2^, ^3^

Patients who ingest cyanide may experience a range of symptoms that can occur within minutes to hours of exposure. In low‐dose exposures, symptoms may include headache, dizziness, confusion, vomiting, nausea, and abdominal cramping. High doses can result in dyspnea, respiratory depression, apnea, hypotension, arrhythmia, coma, and seizure.^3^

Cyanide poisoning diagnosis is difficult, as it presents with nonspecific symptoms. Treatment for cyanide poisoning typically involves supportive care, such as oxygen therapy and intravenous fluid, as well as detoxification and antidotal therapy.^3^

Here, we report a rare case of acute cyanide poisoning with severe metabolic acidosis following the ingestion of bitter almonds. We highlight the importance of timely gastric lavage, decontamination, and antidotal therapy in managing this condition and saving the patient's life.

## 2 CASE PRESENTATION

A 36‐year‐old woman with a history of three suicide attempts and no significant drug history presented to the emergency department of a regional hospital with a chief complaint of vomiting. She ingested approximately 40 bitter almonds in an attempt to commit suicide. The patient possessed an almond orchard containing trees that yielded bitter almonds. Subsequently, she harvested bitter almonds from these particular trees and ingested them in a suicide attempt. She had eaten bitter almond 1 h prior to admission. Vomiting ensued within a 20‐min timeframe. The vomit was non‐hemorrhagic.

Upon admission to the regional hospital, she was confused. The Glasgow Coma Scale (GCS) was 9/15. The initial vital signs showed tachycardia and hypertension as indicated in Table 2. In the physical examination, the skin manifested a normal coloration, and the oral mucosal color was normal. Neurological examination revealed bilateral pupil dilation. Cardiovascular and pulmonary examinations were normal. Two hours after admission, the patient exhibited hypotension as shown in Table 2. Subsequently, after 4 h of admission, the patient became unconscious. Concurrently, blood pressure declined and pulse rate dropped as indicated in Table 2. In response to these developments, intubation was initiated. Owing to the low blood pressure, norepinephrine was administered, resulting in a subsequent increase in blood pressure and pulse rate after 30 min. Venous blood gas analysis at the regional hospital disclosed severe metabolic acidosis (pH = 6.92, pO_2_ = 43 mm Hg, HCO_3_ = 8.6 mmol/L, pCO_2_ = 42.7 mm Hg, base excess = −25.9 mmol/L). Detailed laboratory findings from the regional hospital are provided in Table 1. The lack of a laboratory facility prevented the measurement of blood cyanide levels. Nevertheless, based on clinical and laboratory observations, along with a confirmed history of ingesting bitter almonds, the diagnosis established was acute cyanide poisoning stemming from the oral consumption of amygdalin‐containing food. The electrocardiogram (ECG) exhibited sinus tachycardia. Gastric lavage was conducted, and a singular dose of 60 g (1 g/kg) of activated charcoal was administered. Additionally, an infusion of three vials of sodium bicarbonate (150 mEq) was initiated to address metabolic acidosis. Unfortunately, the regional hospital lacked a specific antidote for cyanide poisoning, necessitating the patient's transfer to the primary referral center for poisoning cases in our province for further management.

**TABLE 1:** Lab data in the regional hospital.

Laboratory tests	Results
WBC	7000 cell/μL (4400–11,000)
Hb	14.2 g/dL (12–16)
BUN	8 mg/dL (7–19)
Neutrophils	32.5% (50–70)
Lymphocytes	63% (20–40)
BS	156 mg/dL (70–135)
RDW	15.7% (11.5–15.5)
P	2 mg/dL (2.6–4.5)
Na	144 mEq/L (138–150)
Cl	105 mEq/L (85–140)
K	2.92 mEq/L (3.8–5)
AST	33 U/L (up to 31)
ALT	10 U/L (up to 31)
pH	6.92 (7.31–7.41)
pCO_2_	42.7 mmHg (41–51)
LDH	574 IU/L (100–480)
HCT	49% (35–45)
CPK	189 μg /L (30–180)
HCO_3_	8.3 mmol/L (23–29)
BE	−25.9 mmol/L (−3/3_ + 3)
Anion gap	30.7 mmol/L (4–12)
INR	1 (1–1.5)
Cr	0.9 mg/dL (0/7–1.3)

Abbreviations: ALT, alanine transaminase; AST, aspartate transaminase; BE, base excess; BS, blood sugar; BUN, blood urea nitrogen; Cl, chloride; CPK, creatine phosphokinase; Cr, creatinine; HB, hemoglobin; HCO_3_, bicarbonate; HCT, hematocrit; INR, international normalized ratio; K, potassium; LDH, lactate dehydrogenase; Na, sodium; P, phosphorous; PCO_2_, partial pressure of carbon dioxide; pH, potential of hydrogen; RDW, red cell distribution width; WBC, white blood cell.

After 7 h, she was transferred to the poisoning referral hospital. Upon arrival at our facility, the patient persisted in an unconscious state with a Glasgow Coma Scale (GCS) of 6/15 and the following vital signs: blood pressure of 90/60 mm Hg, pulse rate of 80 beats/min, oxygen saturation of 97% on mechanical ventilation, and a temperature of 37°C. The electrocardiogram (ECG) displayed a normal sinus rhythm. Blood glucose level was measured at 100 mg/dL (normal range: 70–135 mg/dL). Following clinical consultation with anesthesia services, the patient was admitted to the intensive care unit (ICU). The ventilation settings were configured as follows: Synchronized intermittent mandatory ventilation (SIMV), tidal volume (TV) = 450, rate = 12, pressure support = 12, Fio2 = 60%. Intravenous administration of 300 mg sodium nitrite, a cyanide antidote, was initiated for the patient. However, the patient became hypotensive, prompting the commencement of a norepinephrine infusion at a rate of 10 μg/min (0.1–1 μg/kg/min). Throughout the hospitalization period, 150 mEq sodium bicarbonate infusions were administered due to metabolic acidosis. Unfortunately, sodium thiosulfate 25% was not available in the emergency room for administration. After 4 h of admission to the referral hospital, the venous blood gas pH corrected within the normal range as indicated in Table 2.

**TABLE 2:** Clinical course of presentation and venous blood gas analysis (VBG).

Time after bitter almond ingestion	Level of consciousness, GCS	Intubation	Respiratory rate	Spo_2_	Pulse rate (beats/min)	Blood pressure (mmHg)	pH (7.31–7.41)	pCO_2_ mm Hg (41–51)	pO_2_ mm Hg (30–40)	HCO_3_ mmol/L (23–29)	BE mmol/L (−3/3_ + 3)
1 h	Confused, 9/15	–	16	95%	117	160/85	6.92	42.7	43	8.3	−25.9
3 h	Confused, 9/15	–	22	95%	110	99/60	–	–	–	–	–
5 h	Unconsciousness, 6/15	Intubated	–	95%	104	60/30	–	–		–	–
5 h and 30 min	Unconsciousness, 6/15	Intubated	–	95%	105	80/60	–	–		–	–
8 h	Unconsciousness, 6/15	Intubated	–	97%	80	90/60	7.20	33	36	45	−5.1
12 h	Unconsciousness, 6/15	Intubated	–	95%	73	97/59	7.39	43.7	75.2	26.4	1.3
25 h	Consciousness, 12/15	Extubated	22	100% with mask	86	102/62	7.46	23.9	38	27.9	3.9

Abbreviations: BE, base excess; HCO3, bicarbonate; pCO2, partial pressure of carbon dioxide; pH, potential of hydrogen; pO2, partial pressure of oxygen; Spo2, oxygen saturation.

A day later, the patient became conscious and was successfully extubated the vital signs are shown in Table 2. After 2 days in the referral hospital, the patient was transferred from the intensive care unit (ICU) to the ward. Three days after admission to the referral hospital, she was transferred to a psychiatric center for further care.

## 3 DISCUSSION

In this case report we presented severe cyanide poisoning after bitter almond ingestion that dramatically improved after supportive care and treatment with sodium nitrite. Cyanide exposure represents a significant contributor to morbidity and mortality. A retrospective analysis of 255 cyanide poisoning fatalities in South Korea from 2005 to 2010 showed that the average age of the deceased individuals was 41.88 years, with males constituting the majority of cases. Notably, 97.3% of the fatalities were attributed to suicide.^4^ Over the period from 2008 to 2019, the UK National Poisons Information Service recorded 1252 reports of suspected cyanide poisoning with 239 cases (19%) involving children under the age of 10. The primary sources of exposure were the ingestion of plants (35%) and smoke inhalation (32%). Notably, severe and fatal cases were predominantly associated with smoke inhalation, constituting 71% of such incidents.^5^

Clinical manifestations of cyanide toxicity are nonspecific. Initial symptoms encompass headaches, dizziness, confusion, and vomiting. Progression of the condition may give rise to seizures and diminished levels of consciousness. Early indicators of respiratory and cardiovascular involvement include tachypnea and tachycardia, whereas late‐stage manifestations involve apnea, hemodynamic collapse, and irregular heart rhythms. Approximately 40% of individuals exposed to cyanide may detect the characteristic odor of bitter almonds. A systematic review showed that the cherry red skin and bitter almond odor cannot be reliably considered indicators of cyanide toxicity.^6^

Approximately 67% of patients experiencing cyanide poisoning demonstrate acute metabolic acidosis.^7^ The shift from aerobic to anaerobic metabolism induces substantial lactate production, leading to a notable high anion gap acidosis. The measurement of blood cyanide concentration is imperative for confirming toxicity. However, this assessment is not promptly available enough to impact initial treatment decisions. Cyanide electrocardiography lacks specificity. Reported rhythm disturbances encompass sinus tachycardia, Brady arrhythmias, atrial fibrillation, ventricular tachycardia, and ventricular fibrillation. Additionally, observed ST segment alterations include elevation or depression, shortened ST segments, and fusion of the T wave into the QRS complex.

Cyanide's minimum lethal dose is estimated at 0.5 mg/kg.^8^ The consumption of six to ten bitter almonds can result in severe poisoning, whereas ingesting fifty of them could be fatal for an individual.^9^ The approach to treating cyanide poisoning varies based on factors such as the patient's risk factors for antidote toxicity, the type, and potential severity of the exposure, the progression of clinical symptoms, and their proximity to medical facilities.^10^

In cases of mild poisoning, treatment based on clinical features may involve rest and oxygen. Moderate and severe poisoning, necessitates the use of antidotes.^11^ However, several case reports indicate that supportive care alone has successfully treated patients with severe poisoning. For instance, a reported case involved a patient with severe cyanide poisoning from the subcutaneous injection, who was solely treated with hemodialysis without antidote therapy. In a case series study, nine patients poisoned by cyanide inhalation recovered with supportive care alone, without receiving any antidote.^12^ Supportive care like basic life support (ABCs), bicarbonate for correcting metabolic acidosis, gastric lavage, and activated charcoal. High‐flow oxygen (100%) is crucial, as it synergistically enhances the effects of the antidote. Gastric lavage and the administration of activated charcoal aim to eliminate any remaining cyanide and are considered reasonable measures. It is important to note that activated charcoal has a relatively low binding capacity to cyanide, and one gram of charcoal binds only 35 mg of cyanide.^8^, ^13^ Due to the rapid absorption of cyanide, the administration of activated charcoal and gastric decontamination should be initiated within the initial 1–4 h. Activated charcoal yields optimal effectiveness when administered within the initial hour following ingestion.^14^

In cases of cardiorespiratory collapse accompanied by either a substantial blood cyanide level or apparent signs of cyanide poisoning, it is strongly recommended to administer an antidote.^15^ Various antidotes are available for the treatment of cyanide poisoning, and ongoing research is contributing to the development of new antidotes. The initial category of antidotes includes cobalt compounds. Hydroxocobalamin, a naturally occurring variant of vitamin B12, serves as one such antidote. It replaces the hydroxy group with cyanide, leading to the formation of cyanocobalamin, a nontoxic substance eliminable through the kidneys.^16^ Hydroxocobalamin has not been associated with clinically significant adverse effects, except for isolated allergic reactions, headaches, temporary asymptomatic increases in blood pressure and bradycardia, as well as skin and urine discoloration. Hydroxocobalamin is the preferred treatment for suspected cyanide poisoning as it does not induce hypotension or worsen concerns related to diminished oxygen‐carrying capacity. Dicobalt edetate, commonly used in acute cyanide poisoning, exhibits visible side effects such as hypertension, nausea, vomiting, urticarial, laryngeal edema, anaphylactic shock, hypotension, and arrhythmias, thereby limiting its use.^7^ Sodium thiosulfate, as another antidote for cyanide, functions as a sulfur donor and converts cyanide into a less toxic agent known as thiocyanate.^17^ However, the efficacy of sodium thiosulfate as a remedy is impeded by its delayed onset of action, short duration of effectiveness, and limited distribution within the body.^13^ Many studies have evaluated the effectiveness of sodium thiosulfate when administered in conjunction with other antidotes, such as hydroxocobalamin. It is important to note that thiosulfate is contraindicated for patients with renal insufficiency due to the potential toxicity resulting from the formation of thiocyanate.^16^ The Lily Cyanide Antidote Kit, containing amyl nitrite, sodium nitrite, and sodium thiosulfate, is no longer available.^7^ Nitrites, such as sodium nitrite or amyl nitrite, induce the oxidation of iron in hemoglobin, changing it from ferrous to ferric iron and resulting in the formation of methemoglobin which can then bind cyanide.^8^, ^16^

The efficacy of amyl nitrite inhalation as an initial remedy for cyanide poisoning is a subject of frequent debate due to its limited capacity to generate methemoglobin beyond 6%. Additionally, nitrite induces vasodilation and presents adverse side effects such as hypotension and syncope, restricting its use.^16^ In the absence of hydroxocobalamin, it is advised to administer sodium nitrite for cyanide poisoning.^18^ Sodium nitrite leads to higher methemoglobin levels compared to amyl nitrite, but this increase is associated with problematic low blood pressure. Accurate dosing of sodium nitrite is crucial in children and individuals with anemia to prevent excessive met hemoglobin formation.

## 4 CONCLUSION

In conclusion, supportive care especially gastric lavage is important in cyanide poisoning management. Our patient improved with supportive care including sodium bicarbonate infusion for correcting metabolic acidosis, and sodium nitrite as the antidote. It is concluded that cyanide poisoning can be treated by sodium nitrite monotherapy; when sodium thiosulfate is not available. In the case of ingestion of bitter almonds, performing gastric lavage helps reduce the toxicity.

### 4.1 Limitation of the study

One of the limitations of this case report is the lack of measurement of cyanide serum level, although this test is not available in many hospitals and it is not usually used as a routine test in cyanide poisoning. Another limitation is the lack of evaluating venous blood color and serum lactate level in our patient records.

## AUTHOR CONTRIBUTIONS

**Neda Arabizadeh:** Conceptualization; data curation; investigation; methodology; project administration; resources; supervision; validation; visualization; writing – original draft; writing – review and editing. **Masoud Mahmoudi:** Conceptualization; data curation; investigation; resources; writing – original draft. **Laaya Mokhtar Gandomani:** Data curation; investigation; methodology. **Nastaran Eizadi‐Mood:** Conceptualization; formal analysis; investigation; methodology; project administration; supervision; writing – review and editing.

## FUNDING INFORMATION

This study was not funded.

## ETHICS STATEMENT

This case report has been approved by the Ethical Committee of Isfahan University of Medical Sciences, Isfahan, Iran (Ethical Number: IR.ARI.MUI.REC.1402.137).

## CONSENT

Written informed consent was obtained from the relative of patient to publish this report in accordance with the journal's patient consent policy.

## Data Availability

The data that support the findings of this study are available from the corresponding author upon reasonable request.
